# Effect of irradiance on the emission of short-lived halocarbons from three common tropical marine microalgae

**DOI:** 10.7717/peerj.6758

**Published:** 2019-04-19

**Authors:** Yong-Kian Lim, Fiona Seh-Lin Keng, Siew-Moi Phang, William T. Sturges, Gill Malin, Noorsaadah Abd Rahman

**Affiliations:** 1 Institute of Ocean and Earth Sciences (IOES), University of Malaya, Kuala Lumpur, Malaysia; 2 Institute of Graduate Studies (IPS), University of Malaya, Kuala Lumpur, Malaysia; 3 The Swire Institute of Marine Science and School of Biological Sciences, University of Hong Kong, Hong Kong, SAR; 4 Institute of Biological Sciences, Faculty of Science, University of Malaya, Kuala Lumpur, Malaysia; 5 Centre for Ocean and Atmospheric Sciences, School of Environmental Sciences, University of East Anglia, Norwich, United Kingdom; 6 Department of Chemistry, Faculty of Science, University of Malaya, Kuala Lumpur, Malaysia

**Keywords:** Marine microalgae, Halocarbon emission, Irradiance, Tropics, Environmental change

## Abstract

Marine algae have been reported as important sources of biogenic volatile halocarbons that are emitted into the atmosphere. These compounds are linked to destruction of the ozone layer, thus contributing to climate change. There may be mutual interactions between the halocarbon emission and the environment. In this study, the effect of irradiance on the emission of halocarbons from selected microalgae was investigated. Using controlled laboratory experiments, three tropical marine microalgae cultures, *Synechococcus* sp. UMACC 371 (cyanophyte), *Parachlorella* sp. UMACC 245 (chlorophyte) and *Amphora* sp. UMACC 370 (diatom) were exposed to irradiance of 0, 40 and 120 µmol photons m^−2^s^−1^. Stress in the microalgal cultures was indicated by the photosynthetic performance (F_v_/F_m_, maximum quantum yield). An increase in halocarbon emissions was observed at 120 µmol photons m^−2^s^−1^, together with a decrease in F_v_/F_m_. This was most evident in the release of CH_3_I by *Amphora* sp. *Synechococcus* sp. was observed to be the most affected by irradiance as shown by the increase in emissions of most halocarbons except for CHBr_3_ and CHBr_2_Cl. High positive correlation between F_v_/F_m_ and halocarbon emission rates was observed in *Synechococcus* sp. for CH_2_Br_2_. No clear trends in correlation could be observed for the other halocarbons in the other two microalgal species. This suggests that other mechanisms like mitochondria respiration may contribute to halocarbon production, in addition to photosynthetic performance.

## Introduction

Long-lived anthropogenic substances such as chlorofluorocarbons are widely known as the main cause of the depletion of stratospheric ozone, but more recently, especially since preindustrial times, very short-lived substances, typically of lifetimes no longer than six months, are taking up a more prominent role as the source of stratospheric halogens in affecting the climate, forcing existing photochemical production and loss of stratospheric ozone concentrations (Dorf et al., 2008; Hossaini et al., 2015). Evidence from a recent study showed a continuous decline in the lower stratospheric ozone in the mid-latitude (Ball et al., 2018) in spite of a decrease in ozone depletion in the Antarctic over the past few years (Strahan & Douglass, 2018; Kuttippurath et al., 2013). A balanced upper tropospheric and lower stratospheric chemistry consisting of inorganic bromine-containing compounds (Br_y_) were once thought to be derived entirely from long-lived anthropogenic compounds such as bromomethane (CH_3_Br) and halons (Montzka & Reimann, 2011; Quack et al., 2004). However, these compounds in the stratosphere were lately found to be contributed substantially by short-lived biogenic bromocarbons such as tribromomethane (CHBr_3_) and dibromomethane (CH_2_Br_2_) (Tegtmeier et al., 2012; Fiehn et al., 2017; Ball et al., 2018). An amount of 0.6–3.0 ppt out of the total stratospheric Br_y_ abundance of ∼20–25 ppt of short-lived species are contributed to stratospheric bromine (Hossaini et al., 2016; Carpenter et al., 2014; Dorf et al., 2008; Yang et al., 2005). In convectively active regions, very short-lived halocarbons (VSLHs) including chlorinated and iodinated species get transported up from the ocean surface into the stratosphere during seasonal monsoon (Butler et al., 2007; Fiehn et al., 2017). While the chlorinated VSLHs may affect the distribution of stratospheric ozone, iodinated species such as CH_3_I affects the formation of cloud condensation nuclei and radiation balance, forming new ultra-fine particles and influencing the oxidizing capacity of the atmosphere (Saiz-Lopez et al., 2012). In view of potential adverse impacts of VSLHs on ozone depletion, the need to quantify the emission from different climatic zones is therefore of interest and essential as VSLHs of biogenic sources possess the capability to influence climate by leveraging radiative forcing in the atmosphere. Future increases in their emissions would drive a negative forcing and thereby counterbalance a small fraction of the projected global warming influence due to greenhouse gases (Hossaini et al., 2015).

Of the biogenic sources of VSLHs, marine algae have been the most extensively studied. Previous laboratory and in-situ studies have shown prominent contribution of VSLHs from macro- (seaweeds) and microalgae (phytoplankton) of temperate, polar and tropical regions (Lovelock, Maggs & Wade, 1973; Sturges, Cota & Buckley, 1992; Laturnus, Wiencke & Klöser, 1995; Carpenter & Liss, 2000; Abrahamsson et al., 2004; Keng et al., 2013; Leedham et al., 2013; Leedham Elvidge et al., 2015; Hughes & Sun, 2016; Mithoo-Singh et al., 2017; Lim et al., 2017; Lim et al., 2018; Li et al., 2018). In recent times, more attention has been paid to the biogenic emission from tropical regions due to the prevalence of deep convection due to a combination of high humidity and high insolation (Bergman et al., 2012). The deep convective forces can enhance rapid uplifting of volatile organo-halogenated compounds from the open surface waters into the atmosphere, with the possibility of the climate-active tracers being further distributed in the air through advection to other regions. Whilst macroalgae are a much more significant contributor of organohalogen compounds in the non-upwelling regions of the oceans in terms of biomass, the warm shallow waters of the tropical warm pool are potentially the primary source regions for the biologically-produced halocarbons such as phytoplankton (Mohd Nadzir et al., 2014). Given that marine phytoplankton are widely distributed throughout the euphotic zone of all natural aquatic environments, halocarbon release from algae could be influenced by environmental stresses such as irradiance, nutrient limitation and excess, partial pressure of carbon dioxide (pCO${}_{2}^{\mathrm{sea}}$), temperature and salinity. Reports from studies on the effect of environmental stress on microalgal halocarbon emission from the tropics are scarce in the literature, despite the likely more widespread importance of this phenomenon. In nature, phytoplankton are very likely to experience the continuously fluctuating light levels, and exposure to light more than the usual amount could have a deleterious impact on photosynthetic organisms. For instance, with excess amount of irradiance, photosynthetic efficiency decreased as a result of oxidative damage to the photosynthetic apparatus in temperate microalgae (Hughes et al., 2006; Hughes & Sun, 2016). The deterioration is partly due to a damage to photosystem II (PSII) caused by the oxidation of biochemical compounds like proteins, lipids and pigments by reactive oxygen species (ROS) like hydrogen peroxide (H_2_O_2_) (Hughes et al., 2006; Niyogi, 1999). Depending on the species and standardized experimental conditions, different light intensities may or may not trigger the release of VSLHs from within the cells into the surrounding environment (Hughes et al., 2006; Hughes & Sun, 2016).

Up to the present, only a small number of microalgal incubation studies have reported on the effect of light stress on the emission of VSLHs –of polar (Moore et al., 1996; Hughes & Sun, 2016) and temperate regions (Scarratt & Moore, 1999; Hughes et al., 2006). In general, the latter two studies reported no increase in CH_3_I emission rate from temperate microalgal cultures when exposed to higher irradiance relative to the acclimated lower light control. Moore et al. (1996) revealed a trend of increased concentrations of a brominated compound, CH_2_Br_2_ produced by polar microalgal cultures exposed to a higher level of irradiance. Similarly Hughes & Sun (2016) found a significant positive link between brominating activity of a cold-water marine diatom and short-term changes in photon flux density ranging 0 to 500 µmol photons m^−2^ s^−1^. Report on the contribution and impact of varying irradiance on VSLSs emission from microalgae of tropical origin remains unknown despite such information being needed to improve understanding atmospheric and climate change, as well as allow predictive model development of halogen mechanism as a physiological function in photosynthetic marine organisms. This study represents the first controlled study on the effects of irradiance on the emission of halocarbons from selected tropical marine microalgae, highlighting that the release of VSLHs is species-dependent and compound-specific to the exposure of different irradiance level.

## Materials and Methods

### Experimental design

Three microalgae, the cyanophyte *Synechococcus* sp. UMACC 371, diatom *Amphora* sp. UMACC 370 and the chlorophyte *Parachlorella* sp. UMACC 245, from the University of Malaya Algae Culture Collection (UMACC) were used. The strains represent three different classes of microalgae that are abundant in the local regions and hence are selected as model organisms for the present experiment. Stock cultures of the microalgae were grown in Provasoli Medium (Prov50) (CCMP, 1996), while for *Amphora* sp., 0.01 g L^−1^ silicate (Na_2_SiO_3_.9H_2_O) was added into the medium (Lim et al., 2018). Irradiance level in the growth incubator was maintained between 35–40 µmol photons m^−2^ s^−1^ for all the cultures. Inoculum for the irradiance experiments were prepared by culturing the microalgae (150 mL) in 250 mL conical flasks under axenic conditions in an incubator shaken at 22 × 10^−2^ rcf (PROTECH, Model: GC-1050). Growth was carried out at 25 °C, and supplied with 40 µmol photons m^−2^ s^−1^ from F30T8/D HITACHI Fluorescent lamps (28 W), on a 12 h light:12 h dark cycle. The cultures, along with control flasks containing only the culture medium, were exposed to the different irradiance levels i.e., 0 (dark condition), 40 (control) and 120 µmol photons m^−2^ s^−1^ (higher irradiance) on Day 4. This was done when the cells were in exponential growth phase (Lim et al., 2018), right before the start of next light cycle. All experiments were carried out in triplicate flasks. Before (t_0_) and after (t_1_) the light incubation, 10 mL and 5 mL aliquots of culture were removed from culture flasks to determine chlorophyll a (chl *a*) content and cell density, respectively as described in Section 2.3. In parallel, 60 mL aliquots of medium were removed from the control flasks and cultures, transferred into tubes for centrifugation (2,419 rcf for 10 min), followed by replenishment of 60 mL fresh medium after removal of supernatant. The samples were then transferred to air-tight 100 mL glass syringes and incubated for 4 h (Lim et al., 2018). Four hours after incubation, the culture from each incubation syringe was gently mixed and filtered into a second 100 mL glass syringe through a two-syringe (0.2 µm Merck filter unit) closed filter system to prevent bubble formation in the syringe. This filtrate in the second syringe was sent for halocarbon determination. In order to obtain the net emission, the concentration of halocarbon from the seawater medium control was subtracted from the sample (Table S1). The net halocarbon concentration was normalized to biomass, based on chl *a* (pmol mg^−1^ h^−1^) using 40 mL of the same cultures. The net halocarbon concentration was also normalized to cell density (pmol cell^−1^ h^−1^) to highlight important comparisons of the emission rate. The normalized halocarbon concentrations were used for calculating the emission rates before and after the 12 h of light-exposure. Calculation of emission rate is conducted following Lim et al. (2018):

\begin{eqnarray*}Emission rate= \frac{Concentration of halocarbons}{Biomass\times Incubation time} \end{eqnarray*}

Where:

Emission rate = based on chl *a* (pmol mg^−1^ h^−1^) or cell density (pmol cell^−1^ h^−1^)

Halocarbon concentration = pmol L^−1^

Biomass = chl *a* (mg L^−1^) or cell density (cell mL^−1^)

Time of incubation = 4 h

### Analysis and calibration of halocarbons

A GC-MS (Agilent Technologies 7890A) and purge-and-trap system was used to carry out all halocarbon analyses as described in Lim et al. (2018). Calibrations for all targeted halocarbons, namely iodomethane (CH_3_I), tribromomethane (CHBr_3_), dibromomethane (CH_2_Br_2_), trichloromethane (CHCl_3_) and chlorodibromomethane (CHBr_2_Cl) were conducted with gravimetrically prepared liquid standards (Sigma-Aldrich) in HPLC-grade methanol (Fischer Scientific) injected into the medium.

A five-point calibration curve was used to determine the concentration of halocarbons from both samples and phytoplankton-free controls (See supplementary data, Fig. S1 for calibrations). The regression coefficient (*r*^2^) for the linear calibration curve of all five compounds was above 0.95. Sensitivity drift in the system was corrected based on the internal standards as described in Lim et al. (2018). Detection limits determined by signal-to-noise ratio and measurement of liquid standards injected into purged seawater blanks (Abrahamsson & Pedersén, 2000; Hughes et al., 2006), were on the order of 0.1 pmol L^−1^ for CH_3_I (142 m/z), CHBr_3_ (173 m/z), CHCl_3_ (83 m/z) and CH_2_Br_2_ (174 m/z) and 0.01 pmol L^−1^ for CHBr_2_Cl (129 m/z).

### Cell biomass

Cells were counted using the Improved Double-Neubauer Haemocytometer (Teoh, Phang & Chu, 2013) under a light microscope of 100x total magnification. The culture samples were trapped on Whatmann membrane filters nylon (0.45 µm) in a Millipore filter, for chl *a* determination. Acetone was used for chl *a* extraction under dark conditions and left at 4 degree Celsius overnight in a refrigerator (Vello et al., 2014; Strickland & Parsons, 1968). Optical density (OD) of the extracts in a 700 µL UV fused Quartz glass cuvette was measured at 665 nm, 645 nm and 630 nm using a spectrophotometer (Shimadzu UV Spectrophotometer Model UV1800) after blank correction, and chl *a* determined as follows:

\begin{eqnarray*}Chla \left( mg {L}^{-1} \right) =(Ca\times Va)/(Vc\times 1000) \end{eqnarray*}

where, Ca = 11.6 (OD_665 nm_) −1.31 (OD_645 nm_) −0.14 (OD_630 nm_)

Va = Volume of acetone (mL) used for extraction

Vc = Volume of culture (L)

### Photosynthetic performance (*F*_v_/*F*_m_)

A Water PAM (Pulse Amplitude Modulation) (Walz, Model: WATER-ED) was used to determine *F*_v_/*F*_m_ to indicate stress response prior to and post exposure to light. *F*_v_/*F*_m_ was also taken prior and post the gas-tight incubation, for observing health of the microalgae, as a quality control procedure (Lim et al., 2018). A 15 min dark-adaptation was conducted on all samples before *F*_v_/*F*_m_ measurement.

### Statistical analysis

Factorial-ANOVA was used to test the significant differences of mean chl *a* and cell density (Table S2), halocarbon emissions (Table S3), and halocarbon emissions with *F*_v_/ *F*_m_ (Table S4) values of the three microalgae, under difference irradiance levels. Correlation analysis for emission rates of the halocarbons in terms of chl *a* (Table 1A) and between *F*_v_/*F*_m_ and halocarbon emission rates (Table 1B) of the microalgae with irradiance, was carried out using the Pearson Product-Moment correlation coefficient. Statistica 8.0 Statistics software was used for all analyses.

**Table 1:** Pearson Product-Moment correlation coefficient, *r*, (A) of the emission rate with irradiance; (B) between maximum quantum yields (*F*_v_/*F*_m_) of the microalgae (post-light exposure) and their emission rates.

**(A)**					
	CHBr_3_	CH_3_I	CHCl_3_	CHBr_2_Cl	CH_2_Br_2_
CHBr_3_	1.000	0.660^0.000^	0.808^0.000^	0.842^0.000^	0.898^0.000^
CH_3_I	0.660^0.000^	1.000	0.494^0.009^	0.575^0.002^	0.651^0.000^
CHCl_3_	0.808^0.000^	0.494^0.009^	1.000	0.907^0.000^	0.585^0.001^
CHBr_2_Cl	0.842^0.000^	0.575^0.002^	0.907^0.000^	1.000	0.673^0.000^
CH_2_Br_2_	0.898^0.000^	0.651^0.000^	0.585^0.001^	0.673^0.000^	1.000

(B)									
Compound	***Synechococcus*sp. UMACC 371**			***Parachlorella*sp. UMACC 245**			***Amphora*sp. UMACC 370**		
	**Irradiance level (µmol photons m**^−2^**s**^−1^)								
	**0**	**40**	**120**	**0**	**40**	**120**	**0**	**40**	**120**
CHBr_3_	0.655^0.546^	−0.634^0.563^	−0.500^0.667^	0.500^0.667^	0.963^0.173^	0.373^0.756^	−0.240^0.846^	−0.155^0.901^	0.601^0.590^
CH_3_I	−0.656^0.545^	0.796^0.414^	−0.528^0.646^	−0.495^0.670^	−0.349^0.773^	0.201^0.871^	−0.925^0.248^	0.629^0.567^	0.897^0.292^
CHCl_3_	−0.267^0.828^	0.386^0.748^	0.730^0.480^	0.580^0.606^	−0.499^0.668^	−0.998^0.037^	0.985^0.111^	0.319^0.793^	−0.456^0.699^
CHBr_2_Cl	−0.999^0.017^	0.115^0.927^	−0.233^0.850^	−0.465^0.692^	−0.477^0.683^	−0.874^0.324^	−0.204^0.870^	0.829^0.378^	−0.112^0.929^
CH_2_Br_2_	−0.007^0.995^	0.999^0.029^	−0.626^0.570^	0.732^0.477^	−0.150^0.904^	−0.913^0.267^	−0.565^0.618^	0.056^0.965^	0.821^0.387^

**Notes.**

(a) *n* = 27; (b) *n* = 3; significance level, *p*, is written in superscript next to the correlation coefficient.

## Results

### Growth response

The concentrations of detected VSLHs were normalized to chl *a* to determine the emission rates from the three tropical marine microalgae. Figure S2 shows the concentration of chl *a* after the 12-hour light treatments (dark = 0 µmol photons m^−2^ s^−1^, control = 40 µmol photons m^−2^ s^−1^, higher irradiance = 120 µmol photons m^−2^ s^−1^). All three microalgae showed an increase of chl *a* concentration after incubation. However, in general, the increase in growth was significant (Fig. S2) among different species as well as in different light condition. For example, the highest concentration of chl *a* for *Synechococcus* sp. and *Parachlorella* sp. was from the control group, while *Amphora* sp. had highest chl *a* at the higher irradiance. Compared to the control of the irradiance experiments, chl *a* was observed to decrease when exposed to the high irradiance for *Synechococcus* sp. and *Parachlorella* sp., in contrast to *Amphora* sp. (Fig. S2) which increased. Chl *a* was lowest for all three microalgal species cultured in complete darkness when compared to the control irradiance.

The primary goal of this study is to understand the effect of irradiance on halocarbon emission. Analysis of the emission rates in relation to biomass production, based on chl *a*, may allow the understanding of the metabolic functions of these compounds. A change in halocarbon emission when normalised to chl *a* may indicate effect on photosynthesis and related physiological processes.

### Changes in *F*_v_/*F*_m_

**Figure 1:**
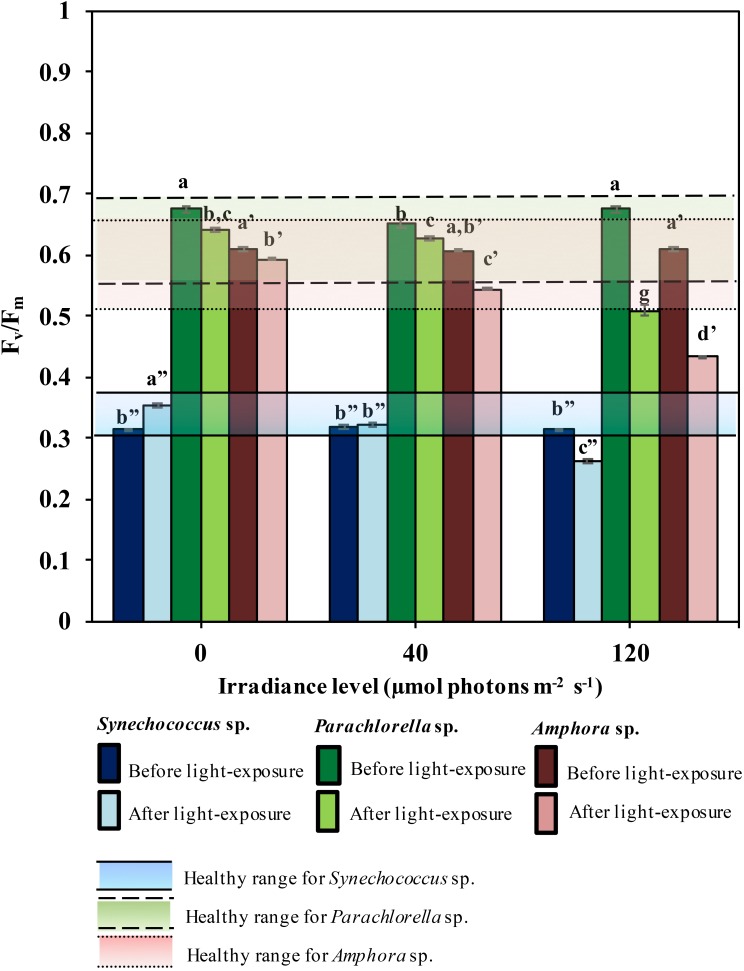
The non-stress range and the changes of maximum quantum yield, F_v_∕F_m_, before and after 12-hour in dark and light-exposure of the three microalgae (*n* = 6) Dashed lines indicate non-stress F_v_∕F_m_ range (upper and lower) of the microalgae (Lim et al., 2018). Vertical error bars denote standard deviation. Different letters indicate significant (*p* < 0.01) differences to compare F_v_∕F_m_ before and after different irradiances in accordance to the three microalgal species as indicated by (’/”). The significance is indicated through homologous grouping using Factorial ANOVA followed by post-hoc Tukey HSD test. (Note: irradiance level of 0 µmol photons m^−2^s^−1^ represents the dark condition).

The maximum quantum yield, *F*_v_/ *F*_m_, which indicates the physiological state (health) of the cells based on changes in photosystem II (PSII) activity in response to environmental change (Hughes et al., 2006; Murchie & Lawson, 2013), is useful to explain the relationship between cell stress and halocarbon production or emission. Figure 1 shows (*n* = 6) *F*_v_/*F*_m_ data before and after the 12-hour light treatment of the three microalgal species. The healthy (non-stress) *F*_v_/*F*_m_ range of upper and lower limit for *Parachlorella* sp., *Synechococcus* sp. and *Amphora* sp. was previously reported at 0.56–0.70, 0.32–0.38 and 0.52–0.67, respectively (Lim et al., 2018). This was used to compare with the range of *F*_v_/ *F*_m_ under irradiance stress in the present study. In general, *F*_v_/*F*_m_ range, including the maximum and minimum values, spanned within the healthy range for control (40 µmol photons m^−2^ s^−1^) and dark (0 µmol photons m^−2^ s^−1^) conditions across the three microalgae. Exposure to irradiance of 120 µmol photons m^−2^ s^−1^, produced *F*_v_/*F*_m_ values within as well as outside the healthy range for the three species.

*F*_v_/*F*_m_ prior to and post 12-hour light treatment for all microalgae is shown in Fig. 1. *F*_v_/ *F*_m_ decreased significantly (Table S3) after incubation under all three irradiances, except *Synechococcus* sp. that showed an increase at 0 µmol photons m^−2^ s^−1^. The calculated decrease (32–40%) in *F*_v_/*F*_m_ in 120 µmol photons m^−2^ s^−1^ is at least ∼3 fold higher than the decrease in the control group (4–12%) and in the dark (3–5%).

### Comparison of halocarbon emissions amongst microalgae

**Figure 2:**
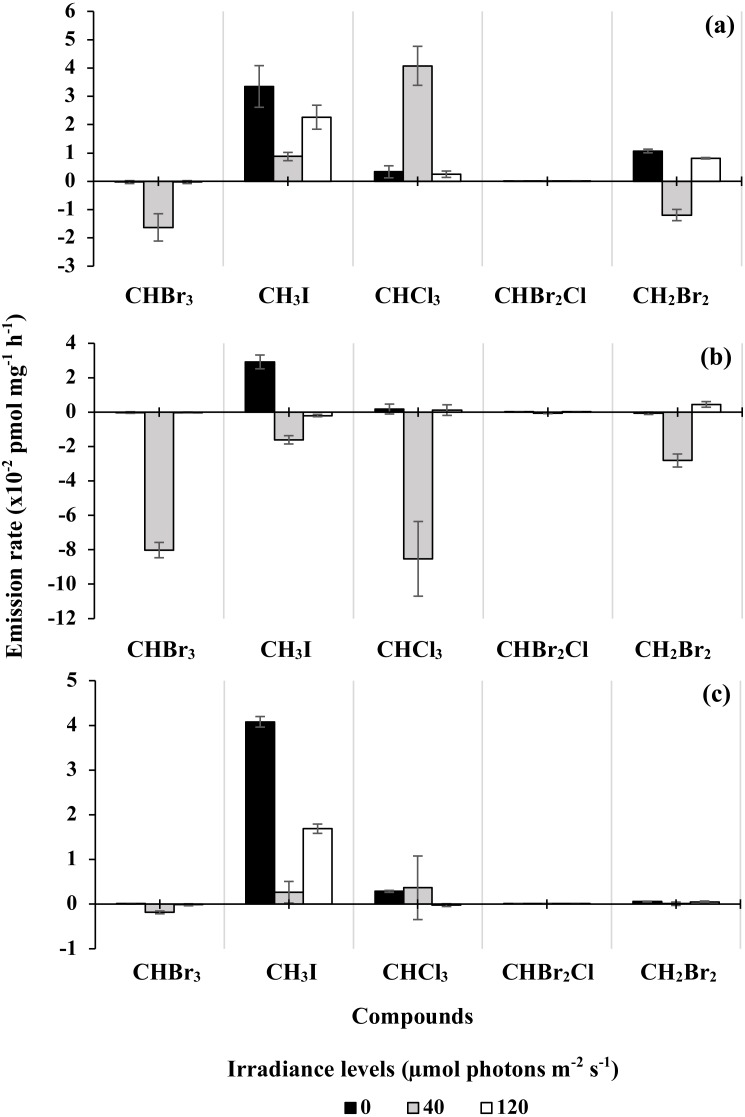
Changes of the five halocarbon emission rates normalized to chlorophyll *a* by (A) *Synechococcus* sp., (B) *Parachlorella* sp., (C) *Amphora* sp. under irradiance levels of 0, 40, 120 µmol photons m^−2^s^−1^. Vertical error bars denote standard deviation from triplicate samples. Positive and negative yields of each compound indicate the increase and decrease of halocarbon production normalized to chl *a*, respectively after irradiance exposure. The significance (*p* < 0.05) of the increase and decrease for each compound is provided in Table S5.

The emission of the five halocarbons measured in triplicates was calculated as a net emission (pmol L^−1^) before and after the 12-hour light treatment as shown in Fig. S3. The increase and decrease in emission are shown by positive and negative values, respectively. For better representation of the effect of irradiance on the halocarbon emissions, the concentration values of the five halocarbons (pmol L^−1^) were normalized to biomass (chl *a*) to calculate the emission rates. Figures 2A–2C (chl *a* normalized) show the increase and decrease (indicated by positive and negative values respectively) of halocarbon emission rates after the light treatments. The significance (*p* < 0.05) of the increase and decrease of each compound can be found in Table S5. Every highlighted red value indicates the exact *p* value of a halocarbon that is significant (*p* < 0.05) to its corresponding halocarbon under specific irradiance level and microalgal species.

In general, an increase of CH_3_I emission was observed for all three microalgae in the dark, but only for *Synechococcus* and *Amphora* at 40 and 120 µmol photons m^−2^ s^−1^ (Fig. S3). However, the emission rate of CH_3_I increased in all microalgae except for *Parachlorella* at 40 and 120 µmol photons m^−2^ s^−1^ (Fig. 2). The emission of CHCl_3_ increased significantly in *Synechococcus* at 40 µmol photons m^−2^ s^−1^ but decreased significantly for *Parachlorella* in the same light condition (Fig. S3). After chl *a* normalization, CHCl_3_ increased in all microalgae except for *Parachlorella* and *Amphora* at 40 and 120 µmol photons m^−2^ s^−1^. Of the brominated compounds, both the emission and emission rate of CH_2_Br_2_ increased for *Synechococcus* in the dark and at higher irradiance (Fig. S3). There was a decrease in CHBr_3_ emission for all three microalgae at all irradiance levels, especially for *Parachlorella* in 40 µmol photons m^−2^ s^−1^ (Fig. S3). There were only minor changes in emissions of CHBr_2_Cl for all microalgae (Fig. S3 and Fig. 2). In Fig. 2A, *Synechococcus* showed an increase in emission rate for CH_3_I, CHCl_3_, and CH_2_Br_2_ after exposure to three different irradiance levels, except CH_2_Br_2_ in the control group (40 µmol photons m^−2^ s^−1^) that showed a decrease in emission rate after the 12-hour exposure. CHBr_3_ showed a decrease in emission rate after exposure to the different irradiance levels.

*Parachlorella* had highest emission rates for all halocarbons before light treatments (Table S6). The increase of CH_3_I emission rate by *Synechococcus* after exposure to higher irradiance (120 µmol photons m^−2^ s^−1^) and in dark (0 µmol photons m^−2^ s^−1^) was at least ∼1.5 times higher than the light control group (Table S6). The increase of emission rate in CHCl_3_ was ∼5 times significantly higher in the control group as compared to other treatment groups of higher irradiance and in the dark. The brominated compound CHBr_3_ showed higher decrease of emission rate in the control group compared with the dark and higher irradiance. The emission rate of CH_3_I by *Synechococcus* was the highest across the three irradiances; the rate ranged between 16.34–17.53 × 10^−3^ pmol mg^−1^ h^−1^ prior to- and between 26.27–49.83 × 10^−3^ pmol mg^−1^ h^−1^ post- light incubation respectively. CHBr_2_Cl was emitted with the lowest emission rate, ranging from 0.00–0.05 × 10^−3^ pmol mg^−1^ h^−1^ before and from 0.14–0.18 × 10^−3^ pmol mg^−1^ h^−1^ after incubation.

The emission rates of all five compounds, CHBr_3_, CH_3_I, CHCl_3_, CHBr_2_Cl and CH_2_Br_2_ for *Parachlorella* decreased after exposure to 40 µmol photons m^−2^ s^−1^. *Parachlorella* had the highest decrease in emission rate of CHCl_3,_ that is 1.76–126.25 × 10^−3^ pmol mg^−1^ h^−1^ (before), and 2.93–41.00 × 10^−3^ pmol mg^−1^ h^−1^, after all light treatments (Table S6). However, after the 12-hour exposure to the dark (0 µmol photons m^−2^ s^−1^), all except the brominated compounds, CHBr_3_ and CH_2_Br_2,_ showed an increase in emission rate. The increase of CH_3_I emission rate in the dark was at least ∼4 times higher than the rest of the compounds. CHCl_3_, CHBr_2_Cl and CH_2_Br_2_ showed an increase while CHBr_3_ and CH_3_I showed the opposite in emission rate after exposure to 120 µmol photons m^−2^ s^−1^. The increase in emission rate of CH_2_Br_2_ by *Parachlorella* after higher light-exposure was twice as high as compared to other four compounds, from 1.30 ± 1.02 × 10^−3^ pmol mg^−1^ h^−1^(before) and 5.78 ± 0.61 × 10^−3^ pmol mg^−1^ h^−1^after light incubation.

All five compounds emitted by *Amphora* showed an increase in emission rate under the three irradiance levels, except CHBr_3_ and CHCl_3_ for higher irradiance exposure and CHBr_3_ in the control group. The increase of CH_3_I emission rate after 12-hour in dark (0 µmol photons m^−2^ s^−1^) was at least ∼2 times higher than the other light treatments (Table S6). Despite the low emission rates of CHBr_2_Cl from *Amphora* before (0.01 ± 0.01 × 10^−3^ pmol mg^−1^ h^−1^) and after (0.10 ± 0.01 × 10^−3^ pmol mg^−1^ h^−1^) dark incubation, the 10 fold increase from this compound was higher as compared to the ∼8 fold increase in CH_3_I emission rate, although emission rates were generally higher for the latter. This trend was also observed for CH_2_Br_2_.

### Correlation analysis of halocarbon emission rates

Table 1A shows the correlation of halocarbon emission rates based on chl *a*. Emission rates of CHBr_3_ was highly-correlated (*r* = 0.842 − 0.898, *p* < 0.01) to other two brominated compounds, CHBr_2_Cl and CH_2_Br_2_. Emission rates of CHCl_3_ was highly correlated (*r* = 0.07, *p*< 0.01) to CHBr_2_Cl. Moderate correlation (*r* = 0.494 − 0.660, *p* < 0.01) was observed for CH_3_I against CHBr_3_, CH_2_Br_2_, CHBr_2_Cl and CHCl_3_.

### Correlation analysis between *F*_v_/*F*_m_ and halocarbon emission rates at different irradiances

The correlation between halocarbon emission rates and maximum quantum yield, *F*_v_/*F*_m_ (after 12 h light treatment), of the microalgae under the different irradiance levels is summarized in Table 1B, normalized to chl *a*. Due to the low number of replicates (*n* = 3), only general observations can be made. In general, the emission rates of brominated compounds from *Synechococcus* and *Parachlorella* were positively correlated to the *F*_v_/*F*_m_ in the dark (0 µmol photons m^−2^ s^−1^). Increasing the irradiance from 0 to 40 µmol photons m^−2^ s^−1^ did not produce much change in the correlations between emission rates and *F*_v_/*F*_m_ for the microalgae, except for *Synechococcus*. The correlation between all halocarbons and *F*_v_/*F*_m_ changed from negative to positive except for CHBr_3_ in *Synechococcus* (Table 1B).

## Discussion

The short-term exposure experiments showed that irradiance affects the emission rates of CHBr_3_, CH_3_I, CHCl_3_, CH_2_Br_2_ and CHBr_2_Cl by the three tropical marine microalgae. To match the light condition where the microalgae had been pre-exposed as stock culture, 40 µmol photons m^−2^ s^−1^ was selected as the control for the present study. 0 µmol photons m^−2^s^−1^ was used as a control for prolonged darkness to compare with 12:12 light:dark cycle that microalgae experience in the tropics. Although higher irradiance level of more than 1,000 µmol photons m^−2^ s^−1^ is usually encountered in the open waters of the tropical region, this study provides useful insight to the capability of the selected microalgae to emit halocarbons after a short-term shift to higher irradiance (120 µmol photons m^−2^ s^−1^) as compared to the control at 40 µmol photons m^−2^ s^−1^. Based on the results from this study, it is thus possible that the microalgae may show even higher emission rate of halogenated compounds, especially CH_3_I with respect to a further decrease in *F*_v_/*F*_m_ upon exposure to a few hundred times higher level of fluctuating irradiance in the natural environment.

Based on the *F*_v_/*F*_m_ results in the present study, the photosynthetic efficiency, *F*_v_/*F*_m_ (Fig. 1) of the three microalgae under higher irradiance level (120 µmol photons m^−2^ s^−1^) were clearly out of the healthy range. This healthy range from the same species has previously been reported by Lim et al. (2018). Note that the healthy range is consistent with the range reported from the control group 40, and 0 µmol photons m^−2^ s^−1^ that describe 12:12 light dark cycle. Therefore, the “out of healthy range” indicates a stress response from the microalgae. This highlights that the microalgae were sensitive to the light changes.

Halocarbon production in the cell is related to stress, in the form of changes in environmental parameters like temperature and irradiance, grazing and microbial attacks, etc. (Leedham et al., 2013; Punitha et al., 2018). It is linked to production of reactive oxygen species (ROS) especially H_2_O_2_ (Nishiyama, Allakhverdiev & Murata, 2006; Serôdio, Vieira & Cruz, 2008). Under high irradiance, light generates oxidative stress resulting in photoinhibition and production of ROS. In photosynthesis, the Mehler Reaction also generates ROS, occurring under “non-stressed” conditions. However, a change in any or combination of environmental parameters, will cause a change in the production of the halocarbons, both in terms of the quantity as well as the composition (Laturnus, Svensson & Wiencke, 2010; Mithoo-Singh et al., 2017).

The halocarbon emission rates normalized to chl *a* were positively correlated (Table 1A). Overall, there were no trends in correlations between *F*_v_/*F*_m_ and the emissions of the five halocarbons under varying irradiance levels across all three microalgal taxa (Table 1B). This indicates that photosynthetic performance may not be a strong influencing factor to the emission of the halocarbons. The total emission is less likely to be affected by difference in growth because the growth of the cultures and the halocarbon emission normalized to chl *a* neither share similar trends nor are consistent when compared under different light intensities in each species (Fig. 2 and Fig. S2). This is further confirmed by the halocarbon emission rate normalized to cell density (Figs. S4 and S5).

*Synechococcus*, the cyanophyte, was observed to be the most affected by irradiance as shown by the increase in emissions of most halocarbons except for CHBr_3_ and CHBr_2_Cl (Fig. 2, Figs. S3 and S5). It could indicate that at higher irradiance and in the dark, *Synechococcus* sp. is easier to be triggered into production of selected halocarbons. Blot et al. (2011) reported that a synergistic effect of light and oxidative stress on PSII photoinactivation in *Synechococcus* sp. was due to the ROS causing both direct damages to the reaction center II and inhibition of the PSII repair cycle. *Synechococcus* and *Prochlorococcus* spp. are known to be sensitive to high irradiance that causes oxidative stress (Mella-Flores et al., 2012). When the two species were exposed to a continuous daily variation of irradiance from 0 to 870 µmol photons m^−2^ s^−1^ on a 12:12 light dark cycles, the *F*_v_/*F*_m_ decreased as the irradiance increased over time. Transcriptional analysis showed that during exposure to high irradiance and UVR, the ocp (orange carotenoid protein) gene was up-regulated; ocp is responsible for dissipation of excessive energy as heat (Mella-Flores et al., 2012). It is possible that the high emission of halocarbons in *Synechococcus*, in the present study where the species was exposed from 0 to 120 µmol photons m^−2^ s^−1^ on a 12:12 light dark cycle, is also another mechanism for dealing with high irradiance stress. There was no clear trend for *Parachlorella*. For *Amphora*, increasing irradiance decreased emissions. This showed that the effect of irradiance on the production of halocarbon is species-dependent and compound-specific.

In the case of exposure to extreme irradiance, example >2,000 µmol photons m^−2^ s^−1^, the *F*_v_/*F*_m_ of the microalgae may fall to below 0.20, much lower than that observed in the present study. This in turn, may drastically reduce halocarbon production as the irradiance received by the microalgae may have passed a threshold for halocarbon production in the cell to occur. The high production of reactive oxidative species (ROS) such as hydrogen peroxide (H_2_O_2_) that acts as a defense mechanism (Nishiyama, Allakhverdiev & Murata, 2006) to cope with high irradiance may be compromised and the microalgae may not be able to recover from the photoinhibition in time to resynthesise the enzymes for recovery. In addition, H_2_O_2_ concentration is insufficient to trigger the production of halocarbon in the cells.

A decrease in photosynthetic efficiency due to oxidative damage to the photosynthetic apparatus could be possibly linked to the emission of other short-lived halocarbons i.e., CHBr_3_, CHCl_3_, CH_2_Br_2_ and CHBr_2_Cl by the three microalgae after exposure to higher irradiance. In the present study, *Amphora* sp. showed an inverse trend of increased CHBr_3_ emission rate and decreased *F*_v_/*F*_m_ after higher irradiance exposure. This could be due to the imbalance between photodamage in photosystem II (PSII) and the repair rate for the damaged PSII caused by the oxidation of lipids, proteins and pigments due to the present oxidative reactive species (ROS); H_2_O_2_ and the hydroxyl radical (OH) induced by higher irradiance (Hughes et al., 2006; Murata & Allakhverdie, 2007). The presence of the ROS, in turn, inactivate the photochemical reactor center of PSII (Murata & Allakhverdie, 2007), which may justify the significant decrease of *F*_v_/*F*_m_ after higher irradiance exposure across the three microalgae, especially *Amphora* sp. in the present study. To further confirm the trend, various tests will be needed to determine the presence of catalytic enzymes such as bromoperoxidases and ROS for possible brominating activity (Lin & Manley, 2012).

Higher production of CH_2_Br_2_ at higher irradiance had been reported by polar *Nitzschia* sp. CCMP 580 and *Porosira glacialis* CCMP 651 cultures (Moore et al., 1996). At low irradiance level (12 µmol photons m^−2^ s^−1^), the emission of CH_2_Br_2_ emitted by both *Nitzschia* sp. and *Porosira glacialis* ranged between 0–380 pmol L^−1^. The emission rate increased to 1300–1600 pmol L^−1^after exposure to higher irradiance level (40 µmol photons m^−2^ s^−1^) over a period of 30 days (Moore et al., 1996). This was consistent with our findings (Fig. 2 and Fig. S5). Higher emission of CH_2_Br_2_ was observed when *Synechococcus* sp., *Parachlorella* sp. and *Amphora* sp. were exposed to higher irradiance. While Moore et al. (1996) reported that there was no clear trend of “higher level of illumination produces higher halocarbon concentration”, this present study showed significant (Table S3) differences in emission rates due to exposure to higher irradiance. The discrepancies could be attributed to different microalgal strains used, the difference in the acclimatization temperature, the exposure duration, and the different range of irradiance level used. Exposure to high irradiance level (20 to 800 µmol photons m^−2^ s^−1^) for 24 h reportedly decreased the CH_3_I emission by *Porphyridium purpureum* (Scarratt & Moore, 1999), while no increase in the emission of iodocarbons by cultures exposed to high irradiance was reported by Hughes et al. (2006). In our study, an increase in CH_3_I emission from *Amphora* sp. and *Synechococcus* sp. was observed, contrary to Scaratt and Moore and Hughes et al.’s reports. This could be due to the difference in the species used and the different geographical zone where the algal species were collected, thus possibly indicating a species-specific response.

The weak correlation between some of the halocarbon emission and *F*_v_/*F*_m_ at different irradiance levels infers that photosynthetic performance may not be a strong influence on emissions in the microalgae. The positive correlation between halocarbon emission rates with *F*_v_/*F*_m_ could be attributed to the increase in H_2_O_2_ from photosynthesis-related activities in the microalgae, below the threshold level resulting in cell stress or membrane destruction, and therefor inhibiting photosynthesis and respiration. This indicates that the halocarbon production may not be influenced strongly by photosynthesis. This is further supported by studies reporting that depletion of nutrients in the culture medium, may or not affect the *F*_v_/*F*_m_ (Cullen, Yang & MacIntyre, 1992; Parkhill, Maillet & Cullen, 2001).

From the percentage of total halide mass (Table 2), *Amphora* sp. emitted the highest amount of halogen and was the highest emitter of iodine as compared to the other two taxa in the present study. This finding was consistent with our earlier findings where halocarbon emission was profiled during microalgal batch culture (Lim et al., 2018).

**Table 2:** Total mass of emitted halides. Total halogen mass emitted as halocarbons and percentage contribution to the total from bromine, chlorine and iodine. Taxa are arranged in decreasing total mass halogens emitted order. Values highlighted in grey, present study; Values not highlighted, reported by Lim et al. (2018).

**Taxa**	**Total halogens emitted (pg)**	**% Br**	**% Cl**	**% I**
*Amphora* sp. UMACC 370	500.5	3.35	9.73	86.92
	5233.6	34.39	5.93	59.7
*Synechococcus* sp. UMACC 371	471.7	14.29	46.88	38.83
	2033.9	35.43	13.40	51.17
*Parachlorella* sp. UMACC 245	98.3	14.59	14.59	70.82
	1573.8	32.29	47.01	21.02

When comparing the present study to the previous growth-cycle (non-light stress) experiment as shown in Table 2, a higher proportion of iodine was emitted by *Parachlorella* sp. by considering the ratios between iodine and bromine, and between iodine and chlorine. There was a change in ratio in the composition of the three halides emitted by *Synechococcus* sp. Despite the short exposure period, these indicate a positive influence of light on the halocarbon emission. Comparing the iodine release between growth stage (Lim et al., 2018) and irradiance experiments, the percentage of iodine contributed by *Amphora* sp. and *Parachlorella* sp. has shown to increase from 60% to 87% and 21% to 71% respectively. Several explanations could be that these two microalgal taxa possess cell structure and size that may be more susceptible to lysis when exposed to higher irradiance, thus releasing more CH_3_I (Hughes, Franklin & Malin, 2011). The possible higher concentration of iodoperoxidase present in these two taxa may also enhance the production of iodine, though the total iodine percentage emitted by *Amphora* sp. was several times higher than *Parachlorella* sp.

## Conclusions

Although the range of irradiance used in the present study is not as large as that occurring under natural conditions, it provides important information for understanding how changes in irradiance may affect halocarbon production under controlled conditions. Based on our results, a change in irradiance varied the production of halocarbons. When microalgae were short-term exposed to three times higher irradiance (120 µmol photons m^−2^ s^−1^) from the normal condition (40 µmol photons m^−2^ s^−1^), a change in halocarbon emission was observed, with a decrease in maximum quantum efficiency (*F*_v_/*F*_m_) from the healthy threshold level, indicating a stress response. To the best of our knowledge, this is the first report that marine microalgae also emit halocarbons when incubated in complete darkness (0 µmol photons m^−2^ s^−1^) under non-stress condition based on *F*_v_/Fm values. Hence, it is important to consider the light and dark cycle, when measuring halocarbon emission, on-site or based on computational modeling, for more accurate quantification of halocarbon emission on a regional or global scale.

*Synechococcus* sp. was shown to be the most sensitive to changes in irradiance as compared to the other two taxa. The emission of CH_3_I by *Amphora* sp. was dominant amongst the five halocarbons, in terms of their total halide mass (pg) and significance. In general, there was no clear similarities in trends between microalgae. This implies that the effect of irradiance on halocarbon emission by microalgae is species-specific. Therefore, a more complete library of halocarbon quantification based on other microalgal species found in local regions is essential to further determine the significance of microalgal-emitted VSLHs in the biogeochemical cycle.

Correlations between halocarbon emission rates and *F*_v_/*F*_m_ were weak across all three taxa. This means that the effects of varying irradiances on halocarbon emission in the three microalgae are not strongly influenced by photosynthetic performance, but could be due to other stress sources that produces H_2_O_2_ or other haloperoxidases. To truly understand and decipher the mechanisms behind halocarbon production as a function of light intensities, other sources such as changes in dissolved organic matter (DOM) composition due to limited and excessive nutrients intra- or extracellularly, mitochondrial respiration or mediation of related halo-enzymes involved in halocarbon production, all of which could possibly elevate the production of halocarbons, should be adequately quantified.

##  Supplemental Information

10.7717/peerj.6758/supp-1 Table S1 Mean concentration (pmol L^−1^) of five halocarbons measured from culture samples and seawater medium controls before (*t*_0_) and after (*t*_1_) exposure of different irradiance levels across three microalgae SD denotes standard deviation. *n* = 3.

10.7717/peerj.6758/supp-2 Table S2 Summary of factorial ANOVA testing the combined effect of irradiance on the growth of *Synechococcus* sp., *Parachlorella* sp. and *Amphora* sp. Data normalized to chl *a* under different irradiance levels of 0, 40 and 120 mmol photons m^−2^s^−1^.

10.7717/peerj.6758/supp-3 Table S3 Summary of factorial ANOVA (univariate) testing the combined effect between halocarbon emission rates and *F*_*v*_∕*F*_*m*_ of *Synechococcus* sp., *Parachlorella* sp. and *Amphora* sp Data normalized to chl *a* under different irradiance levels of 0, 40 and 120 mmol photons m^−2^s^−1^.

10.7717/peerj.6758/supp-4 Table S4 Summary of factorial ANOVA (multivariate) testing the combined effect between halocarbon emission rates with *F*_*v*_∕*F*_*m*_ of *Synechococcus* sp., *Parachlorella* sp. and *Amphora* sp Data normalized to chl *a* under different irradiance levels of 0, 40 and 120 mmol photons m^−2^s^−1^.

10.7717/peerj.6758/supp-5 Table S5 Statistical analysis and significant test for Figs. 1, 2, Figs. S2 and S4

10.7717/peerj.6758/supp-6 Table S6 Mean emission rate ± standard deviation (S.D.) values of the five halocarbons normalized to chl *a* before and after 12-hour of different irradiance levels from the three microalgae (*n* = 3).

10.7717/peerj.6758/supp-7 Figure S1 Calibration curves of peak area against halocarbon concentrations (pmol L^−1^) (a) CHBr_3_, (b) CH_3_I, (c) CHCl_3_, (d) CHBr_2_Cl, (e) CH_2_Br_2_ with their respective linear regression (R^2^)

10.7717/peerj.6758/supp-8 Figure S2 Concentration of chlorophyll *a* (mg L^−1^) after 12-hour light-exposure of the three microalgae under three irradiance levels (0 µmol photons m^−2^s^−1^, 40 µmol photons m^−2^s^−1^ and 120 µmol photons m^-2^ Vertical bars denote standard deviation from triplicate samples. Different letters indicate significant (*p* < 0.01) differences comparing different chl *a* amongst three microalgal species under different irradiances. The significance is indicated through homologous grouping using Factorial ANOVA followed by post-hoc Tukey HSD test.

10.7717/peerj.6758/supp-9 Fgure S3 Changes of halocarbon emission by (a) *Synechococcus* sp. UMACC 371, (b) *Parachlorella* sp. UMACC 245, (c) *Amphora* sp. UMACC 370 under three different irradiance levels, 0, 40, 120 µmol photons m^−2^s^−1^ Vertical bars denote standard deviation from triplicate samples. Positive and negative yields of each compound indicate the increase and decrease of halocarbon production, respectively after irradiance exposure.

10.7717/peerj.6758/supp-10 Figure S4 Concentration of cell density (cell mL^−1^) after 12-hour light-exposure of the three microalgae under three irradiance levels (0µmol photons m^−2^s^−1^, 40 µmol photons m^−2^s^−1^ and 120 µmol photons m[sup]-2[sup Vertical bars denote standard deviation from triplicate samples. Different letters indicate significant (*p* < 0.01) differences comparing different cell density amongst three microalgal species under different irradiances. The significance is indicated through homologous grouping using Factorial ANOVA followed by post-hoc Tukey HSD test.

10.7717/peerj.6758/supp-11 Figure S5 Concentration of cell density (cell mL^−1^) after 12-hour light-exposure of the three microalgae under three irradiance levels (0µmol photons m^−2^s^−1^, 40 µmol photons m^−2^s^−1^ and 120 µmol photons m[sup]-2[sup Vertical bars denote standard deviation from triplicate samples. Different letters indicate significant (*p* < 0.01) differences comparing different cell density amongst three microalgal species under different irradiances. The significance is indicated through homologous grouping using Factorial ANOVA followed by post-hoc Tukey HSD test.

## Additional Information and Declarations

### Data Availability

The following information was supplied regarding data availability: Raw data are available in Table S4 and Fig. S1.
