# Supplementary material for: Effect of irradiance on the emission of short-lived halocarbons from three common tropical marine microalgae
Source: PeerJ. 2019 Apr 19;7:e6758. doi: 10.7717/peerj.6758 (PMC6476285; doi:10.7717/peerj.6758)
Supplement: Table S1 — SD denotes standard deviation. n = 3. [file peerj-07-6758-s001.docx]

Table S1: Mean concentration (pmol L^-1^) of five halocarbons measured from culture samples and seawater medium controls before (t_0_) and after (t_1_) exposure of different irradiance levels across three microalgae.

| **Light level (µmol photons m^-2^ s^-1^)** | **Species** | **Compound** | **Quantifying Ion (m/z)** | **BEFORE 12 light-shock** | | | | **AFTER 12 light-shock** | | | |
| --- | --- | --- | --- | --- | --- | --- | --- | --- | --- | --- | --- |
|  |  |  |  | **Medium control (t_0_)** | | **Samples (t_0_)** | | **Medium control (t_1_)** | | **Samples (t_1_)** | |
|  |  |  |  | **Mean** | **SD** | **Mean** | **SD** | **Mean** | **SD** | **Mean** | **SD** |
| **120** | ***Synechococcus* sp. UMACC 371** | **CHBr_3_** | **173*** | 0.1200 | 0.0017 | 0.1185 | 0.0049 | 0.1485 | 0.0060 | 0.1366 | 0.0096 |
|  |  | **CH_3_I** | **142*** | 0.1467 | 0.0056 | 0.2261 | 0.0083 | 0.1442 | 0.0070 | 0.3811 | 0.0141 |
|  |  | **CHCl_3_** | **83*** | 0.2596 | 0.0092 | 0.2830 | 0.0033 | 0.2223 | 0.0070 | 0.2671 | 0.0044 |
|  |  | **CHBr_2_Cl** | **129*** | 0.0223 | 0.0002 | 0.0225 | 0.0001 | 0.0211 | 0.0002 | 0.0222 | 0.0001 |
|  |  | **CH_2_Br_2_** | **174*** | 0.1176 | 0.0063 | 0.1019 | 0.0076 | 0.0916 | 0.0068 | 0.1414 | 0.0034 |
|  | ***Chlorella* sp. UMACC 245** | **CHBr_3_** | **173*** | 0.1200 | 0.0017 | 0.1094 | 0.0114 | 0.1485 | 0.0060 | 0.1136 | 0.0081 |
|  |  | **CH_3_I** | **142*** | 0.1467 | 0.0056 | 0.1599 | 0.0021 | 0.1442 | 0.0070 | 0.1433 | 0.0022 |
|  |  | **CHCl_3_** | **83*** | 0.2596 | 0.0092 | 0.2601 | 0.0156 | 0.2223 | 0.0070 | 0.2403 | 0.0032 |
|  |  | **CHBr_2_Cl** | **129*** | 0.0223 | 0.0002 | 0.0226 | 0.0001 | 0.0211 | 0.0002 | 0.0219 | 0.0001 |
|  |  | **CH_2_Br_2_** | **174*** | 0.1176 | 0.0063 | 0.1247 | 0.0007 | 0.0916 | 0.0068 | 0.1271 | 0.0033 |
|  | ***Amphora* sp. UMACC 370** | **CHBr_3_** | **173*** | 0.1200 | 0.0017 | 0.1161 | 0.0134 | 0.1485 | 0.0060 | 0.0929 | 0.0065 |
|  |  | **CH_3_I** | **142*** | 0.1467 | 0.0056 | 0.2377 | 0.0067 | 0.1442 | 0.0070 | 0.5949 | 0.0123 |
|  |  | **CHCl_3_** | **83*** | 0.2596 | 0.0092 | 0.2687 | 0.0135 | 0.2223 | 0.0070 | 0.2285 | 0.0059 |
|  |  | **CHBr_2_Cl** | **129*** | 0.0223 | 0.0002 | 0.0221 | 0.0006 | 0.0211 | 0.0002 | 0.0219 | 0.0002 |
|  |  | **CH_2_Br_2_** | **174*** | 0.1176 | 0.0063 | 0.1192 | 0.0068 | 0.0916 | 0.0068 | 0.1043 | 0.0091 |
| **40** | ***Synechococcus* sp. UMACC 371** | **CHBr_3_** | **173*** | 0.2091 | 0.0176 | 0.2981 | 0.0105 | 0.2172 | 0.0197 | 0.2388 | 0.0088 |
|  |  | **CH_3_I** | **142*** | 0.1964 | 0.0175 | 0.2797 | 0.0212 | 0.2027 | 0.0165 | 0.4279 | 0.0108 |
|  |  | **CHCl_3_** | **83*** | 0.8624 | 0.0546 | 0.7000 | 0.7278 | 0.8992 | 0.0035 | 1.2484 | 0.0590 |
|  |  | **CHBr_2_Cl** | **129*** | 0.0247 | 0.0005 | 0.0243 | 0.0003 | 0.0232 | 0.0002 | 0.0245 | 0.0002 |
|  |  | **CH_2_Br_2_** | **174*** | 0.1047 | 0.0039 | 0.1619 | 0.0063 | 0.1645 | 0.0014 | 0.1652 | 0.0016 |
|  | ***Chlorella* sp. UMACC 245** | **CHBr_3_** | **173*** | 0.2091 | 0.0176 | 0.5501 | 0.0202 | 0.2172 | 0.0197 | 0.2300 | 0.0067 |
|  |  | **CH_3_I** | **142*** | 0.1964 | 0.0175 | 0.2715 | 0.0227 | 0.2027 | 0.0165 | 0.2176 | 0.0133 |
|  |  | **CHCl_3_** | **83*** | 0.8624 | 0.0546 | 1.3898 | 0.0478 | 0.8992 | 0.0035 | 1.2162 | 0.0700 |
|  |  | **CHBr_2_Cl** | **129*** | 0.0247 | 0.0005 | 0.0271 | 0.0009 | 0.0232 | 0.0002 | 0.0236 | 0.0003 |
|  |  | **CH_2_Br_2_** | **174*** | 0.1047 | 0.0039 | 0.2225 | 0.0109 | 0.1645 | 0.0014 | 0.1564 | 0.0155 |
|  | ***Amphora* sp. UMACC 370** | **CHBr_3_** | **173*** | 0.2091 | 0.0176 | 0.2376 | 0.0129 | 0.2172 | 0.0197 | 0.1620 | 0.0269 |
|  |  | **CH_3_I** | **142*** | 0.1964 | 0.0175 | 0.2865 | 0.0107 | 0.2027 | 0.0165 | 0.3460 | 0.0337 |
|  |  | **CHCl_3_** | **83*** | 0.8624 | 0.0546 | 1.1152 | 0.0156 | 0.8992 | 0.0035 | 1.2374 | 0.0950 |
|  |  | **CHBr_2_Cl** | **129*** | 0.0247 | 0.0005 | 0.0263 | 0.0006 | 0.0232 | 0.0002 | 0.0263 | 0.0007 |
|  |  | **CH_2_Br_2_** | **174*** | 0.1047 | 0.0039 | 0.1620 | 0.0074 | 0.1645 | 0.0014 | 0.2286 | 0.0017 |
| **0** | ***Synechococcus* sp. UMACC 371** | **CHBr_3_** | **173*** | 0.1200 | 0.0017 | 0.1185 | 0.0049 | 0.1485 | 0.0060 | 0.1158 | 0.0049 |
|  |  | **CH_3_I** | **142*** | 0.1467 | 0.0056 | 0.2261 | 0.0083 | 0.1442 | 0.0070 | 0.4053 | 0.0234 |
|  |  | **CHCl_3_** | **83*** | 0.2596 | 0.0092 | 0.2830 | 0.0033 | 0.2223 | 0.0070 | 0.2648 | 0.0131 |
|  |  | **CHBr_2_Cl** | **129*** | 0.0223 | 0.0002 | 0.0225 | 0.0001 | 0.0211 | 0.0002 | 0.0218 | 0.0001 |
|  |  | **CH_2_Br_2_** | **174*** | 0.1176 | 0.0063 | 0.1019 | 0.0076 | 0.0916 | 0.0068 | 0.1482 | 0.0028 |
|  | ***Chlorella* sp. UMACC 245** | **CHBr_3_** | **173*** | 0.1200 | 0.0017 | 0.1185 | 0.0049 | 0.1485 | 0.0060 | 0.1055 | 0.0043 |
|  |  | **CH_3_I** | **142*** | 0.1467 | 0.0056 | 0.1599 | 0.0021 | 0.1442 | 0.0070 | 0.3401 | 0.0182 |
|  |  | **CHCl_3_** | **83*** | 0.2596 | 0.0092 | 0.2601 | 0.0156 | 0.2223 | 0.0070 | 0.2443 | 0.0052 |
|  |  | **CHBr_2_Cl** | **129*** | 0.0223 | 0.0002 | 0.0226 | 0.0001 | 0.0211 | 0.0002 | 0.0224 | 0.0002 |
|  |  | **CH_2_Br_2_** | **174*** | 0.1176 | 0.0063 | 0.1247 | 0.0007 | 0.0916 | 0.0068 | 0.0957 | 0.0091 |
|  | ***Amphora* sp. UMACC 370** | **CHBr_3_** | **173*** | 0.1200 | 0.0017 | 0.1185 | 0.0049 | 0.1485 | 0.0060 | 0.1485 | 0.0049 |
|  |  | **CH_3_I** | **142*** | 0.1467 | 0.0056 | 0.2377 | 0.0067 | 0.1442 | 0.0070 | 0.9652 | 0.0276 |
|  |  | **CHCl_3_** | **83*** | 0.2596 | 0.0092 | 0.2687 | 0.0135 | 0.2223 | 0.0070 | 0.2825 | 0.0064 |
|  |  | **CHBr_2_Cl** | **129*** | 0.0223 | 0.0002 | 0.0221 | 0.0006 | 0.0211 | 0.0002 | 0.0228 | 0.0001 |
|  |  | **CH_2_Br_2_** | **174*** | 0.1176 | 0.0063 | 0.1192 | 0.0068 | 0.0916 | 0.0068 | 0.1036 | 0.0068 |

SD denotes standard deviation. n = 3.
