# Supplementary material for: Effect of irradiance on the emission of short-lived halocarbons from three common tropical marine microalgae
Source: PeerJ. 2019 Apr 19;7:e6758. doi: 10.7717/peerj.6758 (PMC6476285; doi:10.7717/peerj.6758)
Supplement: Table S2 — Data normalized to chl a under different irradiance levels of 0, 40 and 120 mmol photons m−2s−1. [file peerj-07-6758-s002.docx]

**Supplementary Table S2** Summary of factorial ANOVA testing the combined effect of irradiance on the growth of *Synechococcus* sp., *Parachlorella* sp. and *Amphora* sp.

| Source of variation | SS | Degree of freedom, df | Mean square,  MS | F-ratio | *P* values |
| --- | --- | --- | --- | --- | --- |
|  |  |  |  |  |  |
| *Chlorophyll a (mg/L)* |  |  |  |  |  |
| Intercept | 4.993 | 1 | 4.993 | 2469.105 | 0.000 |
| Species | 0.008 | 2 | 0.004 | 2.025 | 0.161 |
| Light level | 1.638 | 2 | 0.819 | 405.115 | 0.000 |
| Species*Light level | 1.173 | 4 | 0.293 | 145.053 | 0.000 |
| Error | 0.036 | 18 | 0.002 |  |  |
|  |  |  |  |  |  |

Data normalized to chl *a* under different irradiance levels of 0, 40 and 120 μmol photons m^-2^ s^-1^.
