# Supplementary material for: Effect of irradiance on the emission of short-lived halocarbons from three common tropical marine microalgae
Source: PeerJ. 2019 Apr 19;7:e6758. doi: 10.7717/peerj.6758 (PMC6476285; doi:10.7717/peerj.6758)
Supplement: Table S3 — Data normalized to chl a under different irradiance levels of 0, 40 and 120 mmol photons m−2s−1. [file peerj-07-6758-s003.docx]

**Supplementary Table S3** Summary of factorial ANOVA (univariate) testing the combined effect between halocarbon emission rates and F_v_/F_m_ of *Synechococcus* sp., *Parachlorella* sp. and *Amphora* sp.

| Source of variation | SS | Degree of freedom, df | Mean square, MS | F-ratio | *P* values |
| --- | --- | --- | --- | --- | --- |
| *Univariate tests of significance for emission rate of the five halocarbons normalized to chl* a *from the three microalgae under different irradiance levels* | | | | | |
|  | | | | | |
| Species | 0.009 | 2 | 0.005 | 269.557 | 0.000 |
| Light level | 0.010 | 2 | 0.005 | 303.913 | 0.000 |
| Compound | 0.010 | 4 | 0.002 | 145.023 | 0.000 |
| Species*Light level | 0.011 | 4 | 0.003 | 165.062 | 0.000 |
| Species*Compound | 0.005 | 8 | 0.001 | 39.347 | 0.000 |
| Light level*Compound | 0.005 | 8 | 0.001 | 38.586 | 0.000 |
| Species*Light level*Compound | 0.014 | 16 | 0.001 | 50.103 | 0.000 |
|  | | | | | |
| *Univariate tests of significance for F_v_/F_m_ after 12-hour light exposure from the three microalgae under different irradiance levels* | | | | | |
|  | | | | | |
| Species | 1.923 | 2 | 0.961 | 88151 | 0.000 |
| Light level | 0.394 | 2 | 0.197 | 18041 | 0.000 |
| Species*Light level | 0.024 | 4 | 0.006 | 555 | 0.000 |
|  | | | | | |

Data normalized to chl *a* under different irradiance levels of 0, 40 and 120 μmol photons m^-2^ s^-1^.
