# Supplementary material for: Effect of irradiance on the emission of short-lived halocarbons from three common tropical marine microalgae
Source: PeerJ. 2019 Apr 19;7:e6758. doi: 10.7717/peerj.6758 (PMC6476285; doi:10.7717/peerj.6758)
Supplement: Table S4 — Data normalized to chl a under different irradiance levels of 0, 40 and 120 mmol photons m−2s−1. [file peerj-07-6758-s004.docx]

**Supplementary Table S4** Summary of factorial ANOVA (multivariate) testing the combined effect between halocarbon emission rates with F_v_/F_m_ of *Synechococcus* sp., *Parachlorella* sp. and *Amphora* sp.

| Source of variation | Test | Degree of freedom, df | Mean Square, MS | F-ratio | *P* values |  |
| --- | --- | --- | --- | --- | --- | --- |
| *Normalized to chl* a | | | | | | |
| Species | Wilks | 0.000 | 3054 | 4 | 0.000 |  |
| Light level | Wilks | 0.000 | 2061 | 4 | 0.000 |  |
| Compound | Wilks | 0.133 | 39 | 8 | 0.000 |  |
| Species*Light level | Wilks | 0.008 | 230 | 8 | 0.000 |  |
| Species*Compound | Wilks | 0.221 | 13 | 16 | 0.000 |  |
| Light level*Compound | Wilks | 0.224 | 12 | 16 | 0.000 |  |
| *Species*Light level*Compound | Wilks | 0.100 | 12 | 32 | 0.000 |  |
|  | | | | | | |
|  |  |  |  |  |  |  |

Data normalized to chl *a* under different irradiance levels of 0, 40 and 120 μmol photons m^-2^ s^-1^.
