# Supplementary material for: Effect of irradiance on the emission of short-lived halocarbons from three common tropical marine microalgae
Source: PeerJ. 2019 Apr 19;7:e6758. doi: 10.7717/peerj.6758 (PMC6476285; doi:10.7717/peerj.6758)
Supplement: Table S6 [file peerj-07-6758-s006.docx]

Table S6: Mean emission rate ± standard deviation (S.D.) values of the five halocarbons normalized to chl *a* before and after 12-hour of different irradiance levels from the three microalgae. (n = 3).

| **Microalgal cultures** | **Light level (µmol photons m^-2^ s^-1^)** | **Emission rate± S.D. (x10^-3^ pmol mg^-1^ h^-1^)** | | | | | | | | | |
| --- | --- | --- | --- | --- | --- | --- | --- | --- | --- | --- | --- |
|  |  | **Before** | | | | | **After** | | | | |
|  |  | **CHBr_3_** | **CH_3_I** | **CHCl_3_** | **CHBr_2_Cl** | **CH_2_Br_2_** | **CHBr_3_** | **CH_3_I** | **CHCl_3_** | **CHBr_2_Cl** | **CH_2_Br_2_** |
| ***Synechococcus* sp. UMACC 371** | 0 | 0.36±0.61 | 16.93±1.27 | 3.95±1.28 | 0.04±0.03 | 0.03±0.02 | 0.00±0.00 | 49.83±9.12 | 8.14±1.90 | 0.14±0.06 | 10.70±0.65 |
|  | 40 | 18.80±3.34 | 17.53±0.64 | 0.00±0.00 | 0.00±0.00 | 12.03±1.93 | 2.50±1.66 | 26.27±1.01 | 40.77±6.88 | 0.15±0.03 | 0.09±0.13 |
|  | 120 | 0.29±0.51 | 16.34±1.84 | 4.80±1.16 | 0.05±0.02 | 0.00±0.00 | 0.00±0.00 | 38.96±5.53 | 7.28±1.32 | 0.18±0.05 | 8.12±0.26 |
| ***Parachlorella* sp. UMACC 245** | 0 | 0.26±0.45 | 3.16±1.12 | 1.84±2.98 | 0.07±0.04 | 1.42±1.05 | 0.00±0.00 | 31.58±3.12 | 3.54±0.32 | 0.21±0.04 | 0.65±0.40 |
|  | 40 | 81.92±5.39 | 18.01±1.84 | 126.25±14.25 | 0.59±0.31 | 28.29±3.66 | 1.76±1.53 | 1.30±0.59 | 41.00±8.59 | 0.06±0.04 | 0.17±0.30 |
|  | 120 | 0.07±0.13 | 2.41±0.94 | 1.76±3.04 | 0.06±0.06 | 1.30±1.02 | 0.00±0.00 | 0.42±0.72 | 2.93±0.61 | 0.13±0.03 | 5.78±0.61 |
| ***Amphora* sp. UMACC 370** | 0 | 0.08±0.14 | 5.54±0.45 | 0.58±0.31 | 0.02±0.01 | 0.13±0.06 | 0.10±0.17 | 46.02±1.48 | 3.38±0.43 | 0.10±0.01 | 0.67±0.41 |
|  | 40 | 1.85±0.31 | 5.85±0.76 | 16.38±4.33 | 0.11±0.04 | 3.72±0.57 | 0.00±0.00 | 8.50±1.70 | 20.03±5.66 | 0.19±0.04 | 3.80±0.20 |
|  | 120 | 0.15±0.22 | 5.24±0.63 | 0.53±0.27 | 0.01±0.01 | 0.10±0.05 | 0.00±0.00 | 22.10±0.98 | 0.30±0.08 | 0.04±0.02 | 0.62±0.18 |
