# Supplementary figures and images for: Effect of irradiance on the emission of short-lived halocarbons from three common tropical marine microalgae
Source: PeerJ. 2019 Apr 19;7:e6758. doi: 10.7717/peerj.6758 (PMC6476285; doi:10.7717/peerj.6758)

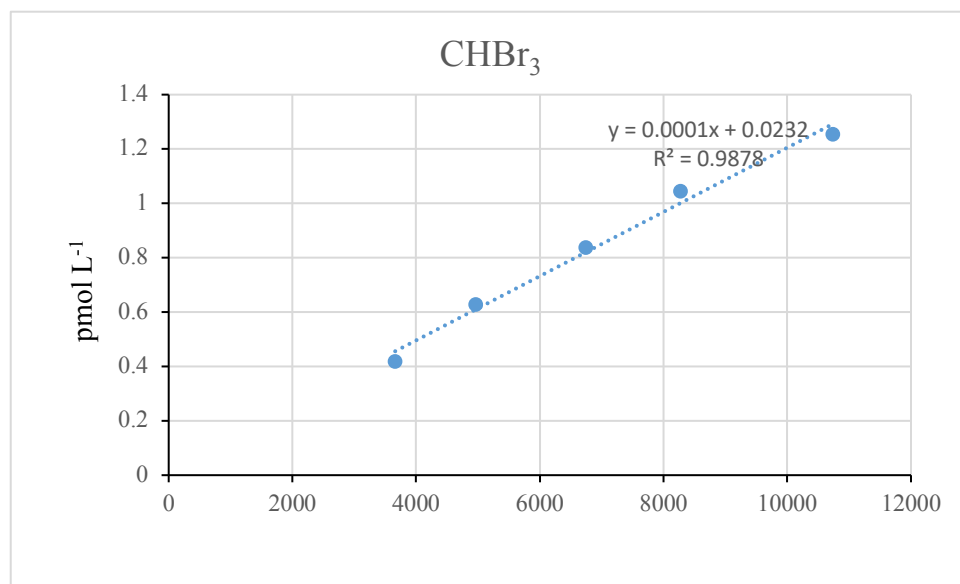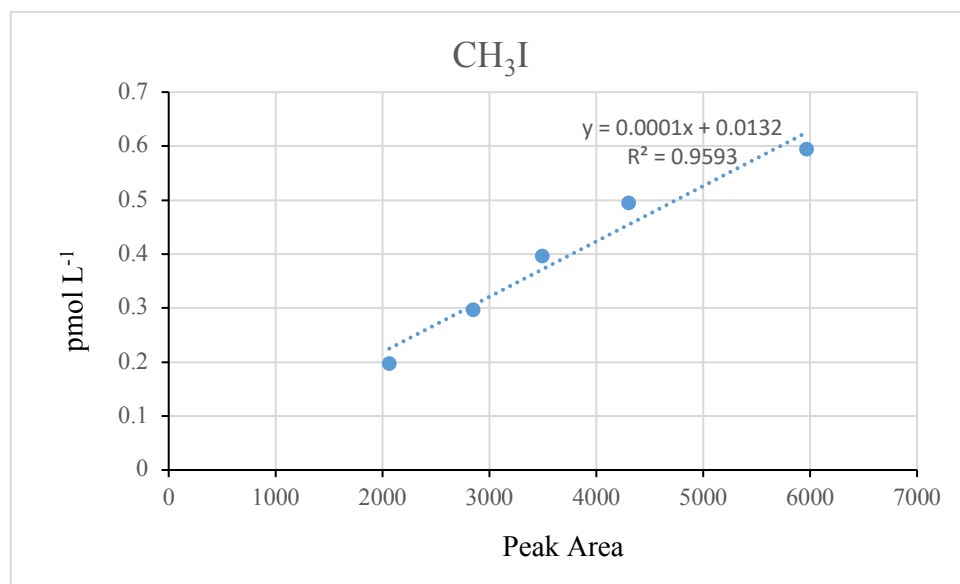

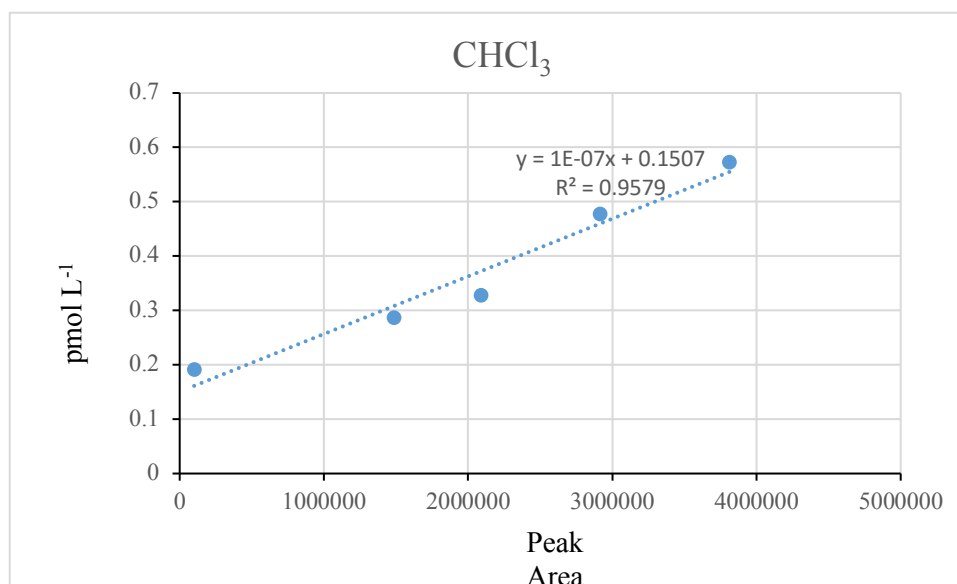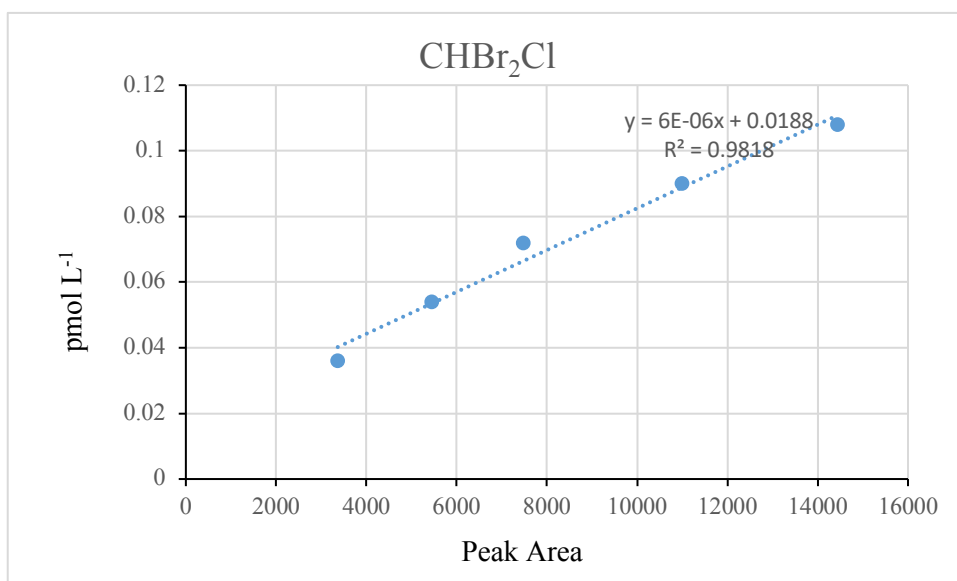

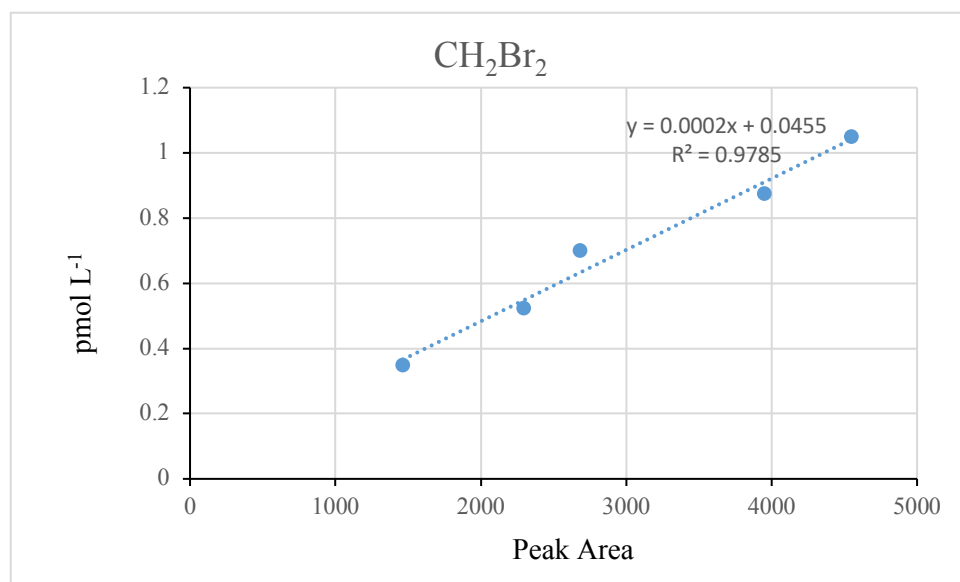

Supplement: Figure S1 [file peerj-07-6758-s007.pdf]

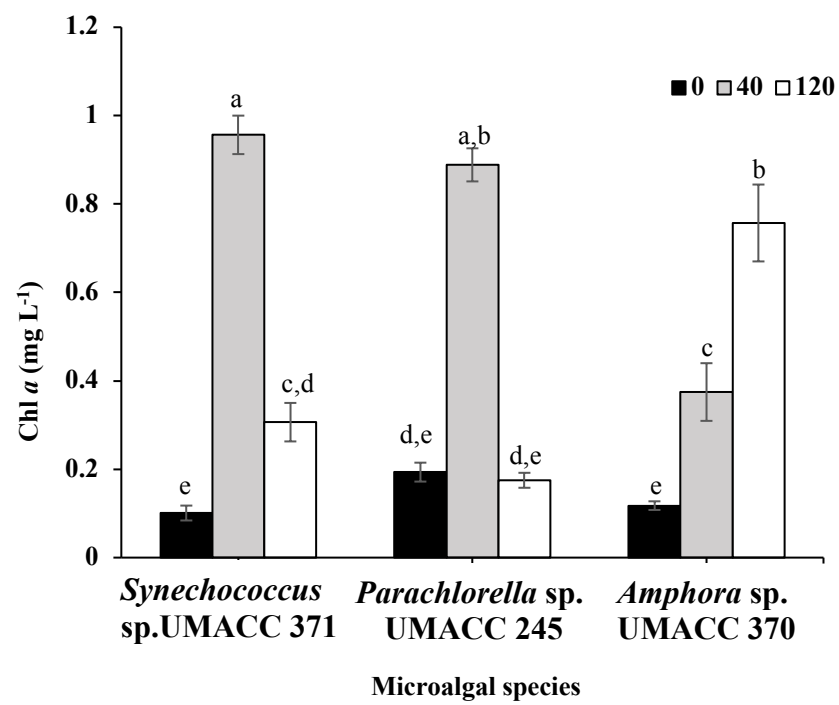

Supplement: Figure S2 [file peerj-07-6758-s008.pdf]

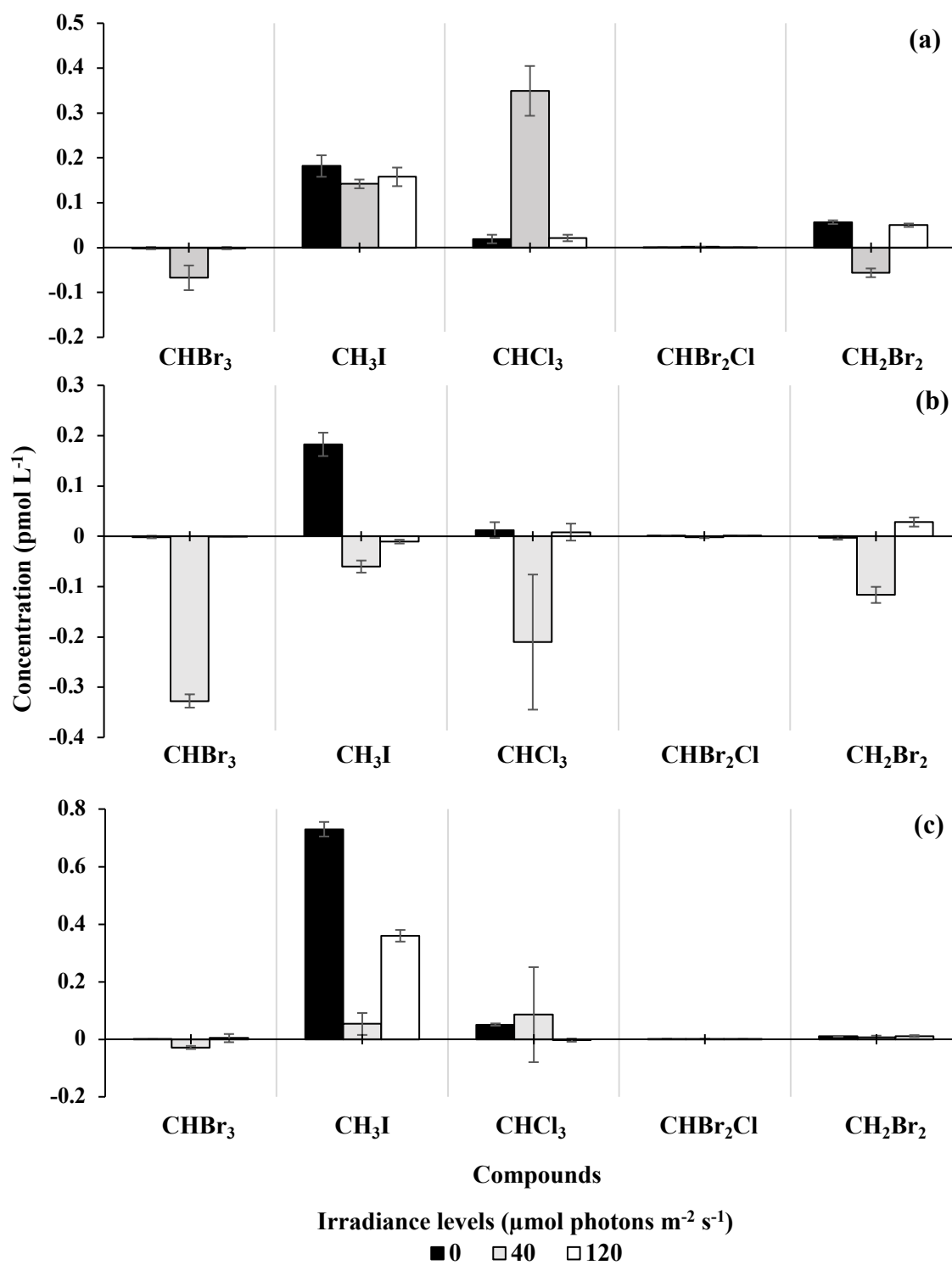

Supplement: Fgure S3 — Vertical bars denote standard deviation from triplicate samples. Positive and negative yields of each compound indicate the increase and decrease of halocarbon production, respectively after irradiance exposure. [file peerj-07-6758-s009.pdf]

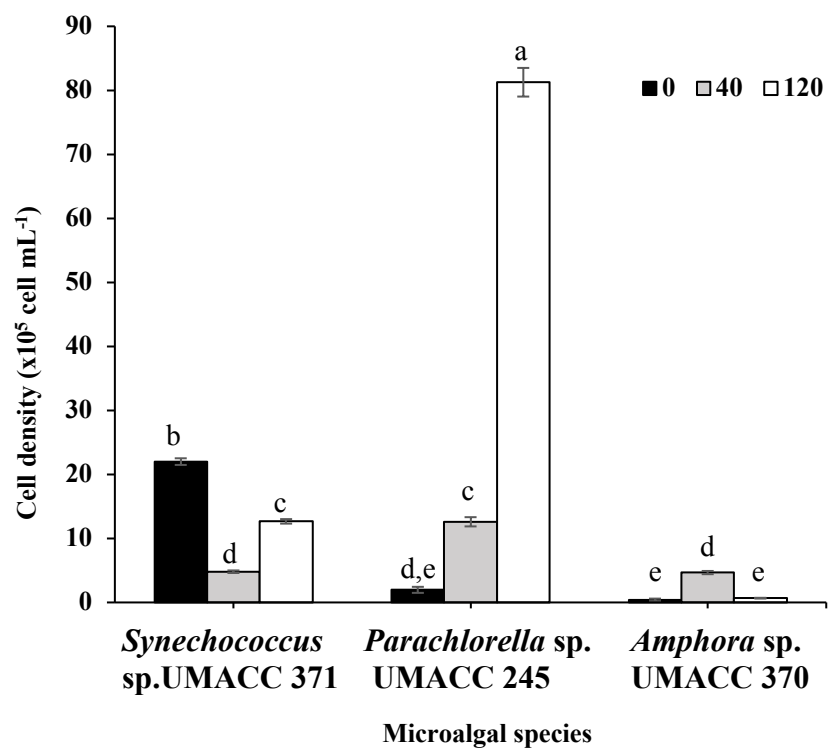

Supplement: Figure S4 — Vertical bars denote standard deviation from triplicate samples. Different letters indicate significant (p < 0.01) differences comparing different cell density amongst three microalgal species under different irradiances. The significance is indicated through homologous grouping using Factorial ANOVA followed by post-hoc Tukey HSD test. [file peerj-07-6758-s010.pdf]

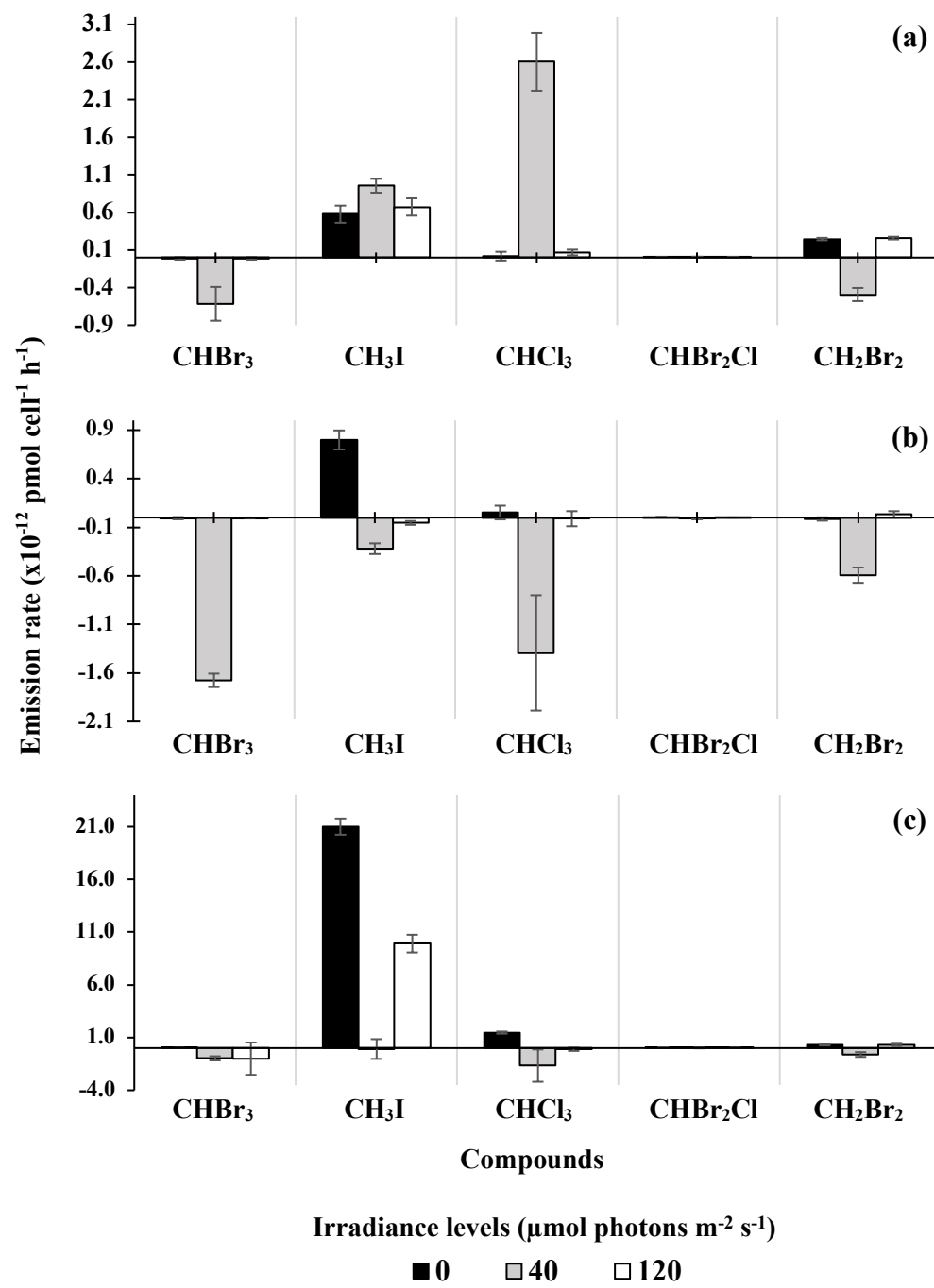

Supplement: Figure S5 — Vertical bars denote standard deviation from triplicate samples. Different letters indicate significant (p < 0.01) differences comparing different cell density amongst three microalgal species under different irradiances. The significance is indicated through homologous grouping using Factorial ANOVA followed by post-hoc Tukey HSD test. [file peerj-07-6758-s011.pdf]
